# Friend or foe: differential responses of rice to invasion by mutualistic or pathogenic fungi revealed by RNAseq and metabolite profiling

**DOI:** 10.1038/srep13624

**Published:** 2015-09-08

**Authors:** Xi-Hui Xu, Chen Wang, Shu-Xian Li, Zhen-Zhu Su, Hui-Na Zhou, Li-Juan Mao, Xiao-Xiao Feng, Ping-Ping Liu, Xia Chen, John Hugh Snyder, Christian P. Kubicek, Chu-Long Zhang, Fu-Cheng Lin

**Affiliations:** 1 State Key Laboratory of Rice Biology, Institute of Biotechnology, Zhejiang University, Hangzhou, 310058, China; 2 Zhengzhou Tobacco Research Institute of CNTC, Zhengzhou 450001, China; 3 Austrian Center of Industrial Biotechnology (ACIB), c/o Vienna University of Technology, 1060 Vienna, Austria

## Abstract

The rice endophyte *Harpophora oryzae* shares a common pathogenic ancestor with the rice blast fungus *Magnaporthe oryzae*. Direct comparison of the interactions between a single plant species and two closely-related (1) pathogenic and (2) mutualistic fungi species can improve our understanding of the evolution of the interactions between plants and fungi that lead to either mutualistic or pathogenic interactions. Differences in the metabolome and transcriptome of rice in response to challenge by *H.* or *M. oryzae* were investigated with GC-MS, RNA-seq, and qRT-PCR. Levels of metabolites of the shikimate and lignin biosynthesis pathways increased continuously in the *M. oryzae*-challenged rice roots (Mo-roots); these pathways were initially induced, but then suppressed, in the *H. oryzae*-challenged rice roots (Ho-roots). Compared to control samples, concentrations of sucrose and maltose were reduced in the Ho-roots and Mo-roots. The expression of most genes encoding enzymes involved in glycolysis and the TCA cycle were suppressed in the Ho-roots, but enhanced in the Mo-roots. The suppressed glycolysis in Ho-roots would result in the accumulation of glucose and fructose which was not detected in the Mo-roots. A novel co-evolution pattern of fungi-host interaction is proposed which highlights the importance of plant host in the evolution of fungal symbioses.

Interactions between plants and fungi span a broad continuum from pathogenic to mutualistic^1^. Whereas asymptomatic fungal endophytes exemplify the mutualistic or commensalistic region of this spectrum, other fungi are virulent pathogens that kill their host plant; still others strongly reduce plant performance and fitness^2^,^3^,^4^. As sessile organisms, plants are unable to escape attack by parasites, so strong defense mechanisms are needed for them to effectively respond to and manage pathogens^5^. Plants also engage in mutualistic interactions with beneficial microorganisms such as root-associated fungi to extend access to nutrients^6^. Plants can tune their physiological responses to prevent detrimental interactions or to support advantageous interactions.

The commonalities and differences between pathogenic and symbiotic colonization strategies of various fungi have been investigated in many studies^7^,^8^,^9^,^10^. It has been reported that both pathogenic and mutualistic interactions follow similar developmental programs, progressing from host identification through to plant cell penetration and re-differentiation of the host cells to establish intracellular interfaces for the exchange of nutrients and information signals^10^,^11^. For example, effector proteins that suppress defense responses and reprogram host cells, were detected in both pathogenic and mutualistic fungi^10^. Separate studies of beneficial arbuscular mycorrhiza (AM) fungi in legumes and rice, and *Phytophthora* pathogens in potatoes and tomatoes, have shown that similar steps occur during the establishment of the interaction in both cases^10^. Furthermore, some fungal endophyte species have been shown to be closely related to phytopathogenic fungal species^12^. Endophytism is evolutionarily transient, with endophytic lineages frequently transitioning to and from pathogenicity^12^,^13^. There are two major groups of endophytes: the clavicipitaceous and the non-clavicipitaceous. Clavicipitaceous endophytes have been proven to have arisen from insect-parasitic ancestors^14^. There is agreement that non-clavicipitaceous endophytes are a polyphyletic group^6^, indicating that the endophytism has originated independently several times. We showed previously that *Harpophora oryzae,* a non-clavicipitaceous endophyte of rice, originates from a common phytopathogenic ancestor of rice blast fungus *Magnaporthe oryzae*^9^. It seems likely that pathogenic and mutualistic interactions, at least in some taxonomic groups, may have arisen from a single host. Therefore, direct comparison of pathogenic and mutualistic interactions in the same plant species should deepen our understanding of the evolution of the particular interactions between plants and fungi that lead to either mutualism or disease.

Knowledge gained from the study of several pathosystems can be used to illustrate typical interactions between host plants and fungal pathogens. Examples include the hemi-biotrophic fungal interactions between grass hosts and *M. oryzae*^15^, obligate biotrophic fungal interactions between barley and powdery mildew^16^, and neoplastic smut disease of maize caused by *Ustilago maydis*^17^. Examples of mutualistic plant-fungi interactions include arbuscular mycorrhizal symbiosis with *Glomeromycota*^18^ as well as endosymbioses with the fungal endophytes *Piriformospora indica*^19^ and *Epichloë/Neotyphodium*^20^. However, the comparison of different types of plant-fungi interactions in the same plant species is hampered by the traditional empirical separation of plant pathology systems and plant-fungi mutualism systems in different plant species^10^. Thus, it would be very valuable to conduct pathology and mutualism experiments with a single plant species, thereby enabling direct empirical comparison between pathogenic and mutualistic plant-fungi interactions. However, to date, there have been few studies that directly compared the responses of one plant species to these two separate types of plant-microbe interactions.

We have recently established an experimental system to evaluate mutualistic and pathogenic interactions with a single host plant (*Oryzae sativa* with *H.* and *M. oryzae*). A comparative genomic and transcriptomic analysis has shown the differential responses of the endosymbiont and the pathogen in their respective interactions with rice, and thus revealed critical components of the evolution of an endophyte from a pathogenic ancestor^9^. It is well known that the re-programming of the metabolisms of the host is fundamentally important in these interactions^8^. However, any differences in the response of rice plants to these two different fungi are as yet unknown. In the biotrophic phase of interactions between rice leaves and *M. oryzae*, there is a flow of nutrients from the host to pathogen that is known to include glucose and fructose^21^. Interestingly, in the biotrophic phase, the glucose and fructose content in rice roots was not affected by *M. oryzae*, while the glucose and fructose content increased significantly in the *H. oryzae*-challaged rice roots^9^. These findings indicate that there are different forms of metabolic re-programming in these interactions. In this study, we investigated the metabolome and the transcriptome of rice in response to inoculation with both *H.* and *M. oryzae*, with the goal of identifying differential plant responses and thus deepening our understanding of the evolution of the interactions between host plants and fungi that lead to either mutualistic or pathogenic interactions.

## Results

### Changes in the rice root metabolome

**Figure 1:**
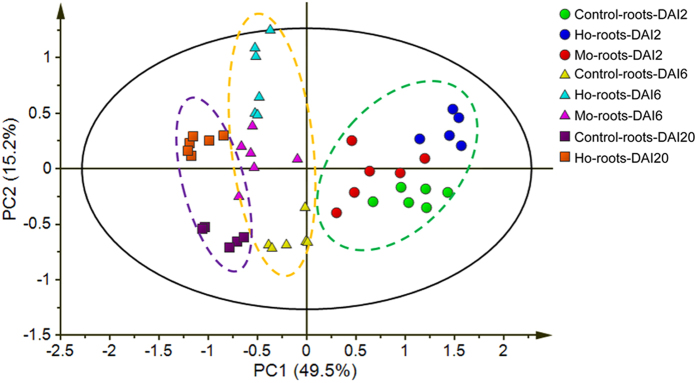
PCA analysis of metabolite profiling data. Scores plot of principal components analysis of rice roots infected with *H. oryzae* (Ho-roots-DAI2, Ho-roots-DAI6, and Ho-roots-DAI20), *M. oryzae* (Mo-roots-DAI2 and Mo-roots-DAI6), or sterile water (Control-roots-DAI2, Control-roots-DAI6, and Control-roots-DAI20) at 2, 6, and 20 days after inoculation (DAI). PC1 and PC2: principal component 1 and principal component 2. Each point represents a metabolite profile of a biological replicate.

GC-MS analysis was used to evaluate Ho-roots at 2, 6, and 20 days after inoculation (DAI), Mo-roots at DAI2 and DAI6, and control roots (Control-roots). By comparing the mass spectra of analyte peaks with those of commercial reference standard compounds, a total of 58 sample metabolites were identified (Supplementary Fig. 1; Supplementary Table S1; Supplementary Table S2). In our principal component analysis (PCA) of all samples, the first two PCs accounted for 64.7% of the total variance of the data, and differentiated among developmental stages (PC1) as well as between fungi (both *H.* and *M. oryzae*) and the control samples (PC2, Fig. 1), suggesting that the majority of the variance in the data resulted from the treatments. Two days after the initial inoculation, there was some degree of overlap in the clustering of the Ho-root and Mo-root samples in the PCA scores plot (Fig. 1). However, samples within each treatment clustered tightly, and different treatments were discriminated at subsequent stages (DAI6, Fig. 1), clearly showing the effect of the different species of fungi on the roots. The profiles indicated a bigger discrepancy in metabolic activity between the Ho-roots and Control-roots than between the Mo-roots and Control-roots (Fig. 1), at both the 2 and 6 day time points.

**Figure 2:**
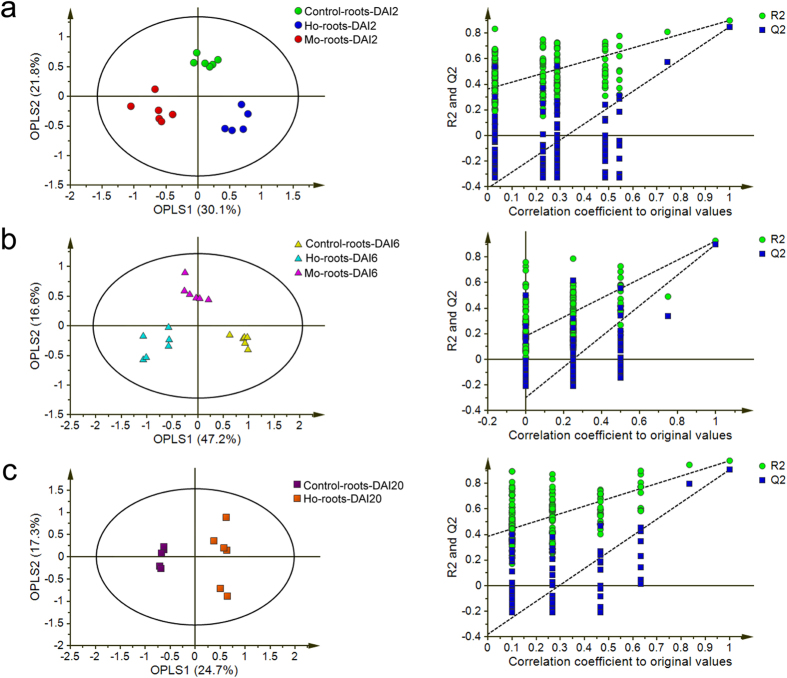
OPLS-DA scores plots (left) and permutation tests (right) of PLS-DA models. The analysis was based on metabolite profiling data of rice roots infected with *H. oryzae* (Ho-roots), *M. oryzae* (Mo-roots), or sterile water (Control-roots) at 2 (**A**), 6 (**B**), and 20 (**C**) days after inoculation. The permutation tests were carried out with 200 random permutations.

In order to further understand the differences between the roots challenged by the different fungi, orthogonal partial least-squares-discriminant analysis (OPLS-DA) models were generated. The OPLS-DA scores plots showed a significant clustering of the rice roots that received different treatments (Control-roots, Ho-roots, and Mo-roots) (Fig. 2). In the OPLS-DA models of the fungi-challenged roots at DAI2, DAI6, and DAI20, the first two components described 90.6%, 90.5%, and 98.5% of the variation and predicted 79.4%, 84.3%, and 96.9% for each infection stage, respectively, according to cross-validation. Furthermore, permutation tests were performed with PLS-DA models to validate each OPLS-DA model, and these tests showed that all of the Q2 and R2 values were higher in the permutation tests than in the OPLS-DA models (Fig. 2). These results demonstrated both high goodness of fit and high predictive capability for the OPLS-DA models. Therefore, we concluded that using the first two components to examine the rice root metabolite profiling results was sufficient.

**Figure 3:**
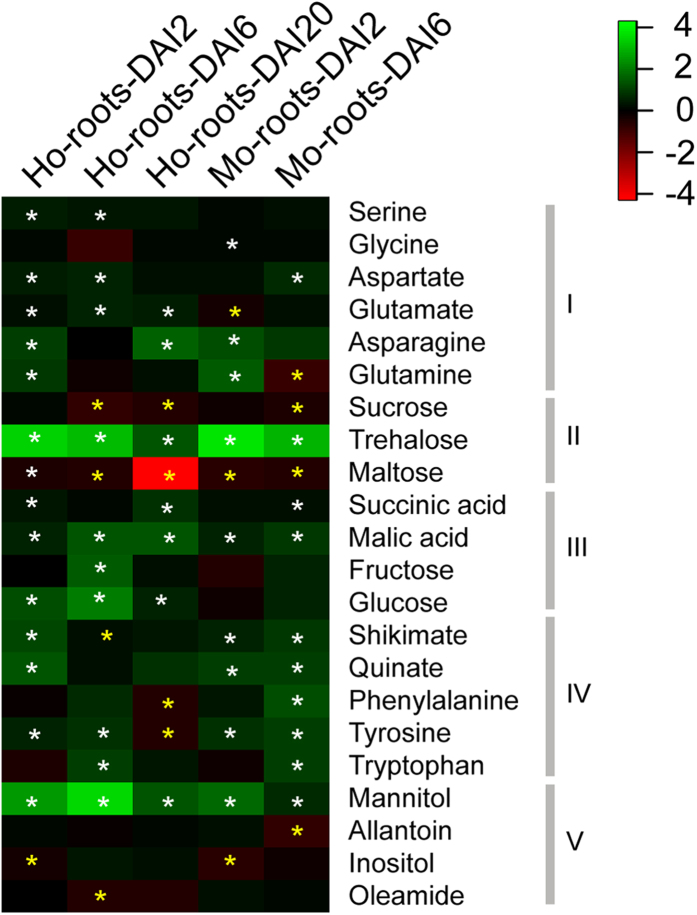
Identification of differentially accumulated metabolites. The most significant metabolites influencing the separation in the OPLS-DA models of rice roots of different treatments are listed. Transcript fold-changes (log2) of Ho-roots vs. Control-roots samples at DAI2, 6 and 20 are indicated. Green, increase in the abundance; red, decrease in the abundance. Asterisks refer to the highly accumulated (white) and reduced (yellow) metabolites with statistical significant changes compared with control determined using a Student’s t-test (P<0.05). I, amino acids; II, central carbon metabolism; III, TCA/glyoxylate cycle; IV, shikimate pathway and aromatic amino acids; V, others. Ho-roots-DAI2, Ho-roots-DAI6, and Ho-roots-DAI20 refer to DEGs of the *H. oryzae*-challenged roots at 2, 6, and 20 days after inoculation, respectively. Mo-roots-DAI2 and Mo-roots-DAI6 refer to DEGs of the *M. oryzae*-challenged roots at 2 and 6 days after inoculation, respectively.

In OPLS-DA modeling, the contribution of each metabolite to OPLS1 and OPLS2 is computed, and each metabolite is given a loading (weighted value) for both OPLS1 and OPLS2. By combining the VIP (variable importance in the projection) values in the loadings plot (Supplementary Fig. S2), metabolites with VIP >1 were selected as differentially accumulated metabolites. Twenty-two such compounds were thusly identified (Fig. 3), and Student’s t-tests showed that these metabolites were significantly different at least between one treatment and the corresponding control samples (p < 0.05). 6 metabolites were differentially accumulated by *H.* and *M. oryzae* at DAI2; and similarly, 11 differentially accumulated metabolites were identified at DAI6 (Fig. 3). The greater number of differentially accumulated metabolites at DAI6 compared to DAI2 was consistent with an observed overlap of the clusters for the Ho-root and Mo-root samples at DAI2 in the PCA analysis.

Three of the twenty-two differentially accumulated metabolites were the disaccharides sucrose, maltose, and trehalose (Fig. 3). In addition, four intermediates of glycolysis and the TCA cycle including succinate, malic acid, fructose, and glucose were detected as differentially accumulated metabolites (Fig. 3). The aromatic amino acids phenylalanine, tyrosine, and tryptophan, together with quinate and shikimate, two precursor compounds in aromatic amino acid biosynthesis, were identified as differentially accumulated metabolites (Fig. 3). In general, the differentially accumulated metabolites were associated with a small number of distinct areas of metabolism, with carbohydrate metabolism and aromatic amino acid metabolism predominating.

### Identification of differently expressed genes (DEGs)

It has been shown that the infection process of rice roots by *H.* and *M. oryzae* proceed with a similar infection strategy at an early stage (DAI2) but clearly differentiate at the middle stage (DAI6)^9^,^22^,^23^. Furthermore, metabolic profiling showed that rice roots responded more similarly to *H.* and *M. oryzae* infection at DAI2 than at DAI6. Thus, in this study, we focused on the differences in the response of rice roots to *H.* and *M. oryzae* infection at the middle and later stages as compared to an early stage, in order to identify gene expression patterns that changed between these infection stages.

**Figure 4:**
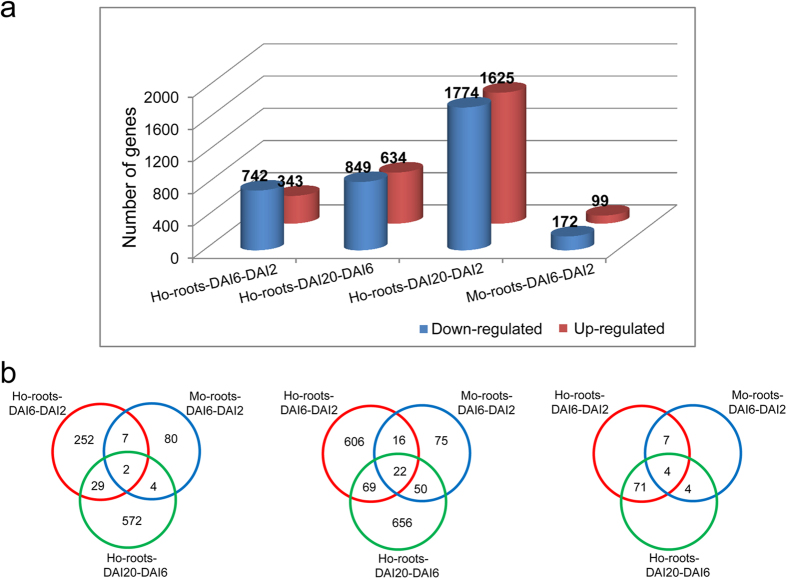
Differentially expressed genes (DEGs) of *H.* and *M. oryzae*-challenged rice roots at different colonization stages. (**a**) The tallies of up-regulated and down-regulated genes. Pair-wise comparisons were made between sample groups. (**b**) Venn diagrams of DEGs. Left, up-regulated genes; middle, down-regulated genes; right, nonuniformly regulated genes. Ho-roots-DAI2, Ho-roots-DAI6, and Ho-roots-DAI20 refer to DEGs of the *H. oryzae*-challenged roots at 2, 6, and 20 days after inoculation, respectively. Mo-roots-DAI2 and Mo-roots-DAI6 refer to DEGs of the *M. oryzae*-challenged roots at 2 and 6 days after inoculation, respectively.

Consequently, RNA isolated from the roots of Ho-roots-DAI2, Ho-roots-DAI6, Ho-roots-DAI20, Mo-roots-DAI2, and Mo-roots-DAI6 (most of the roots infected by *M. oryzae* were dead at DAI20) were evaluated with RNA-seq analysis. Three biological replicates were prepared and analyzed for each sample. In total, 121.71, 109.81, and 102.93 million reads were uniquely mapped to the rice genome sequence (*O. sativa* Japonica Group cultivar Nipponbare; ENSEMBL release 6.12) for the Ho-roots-DAI2, Ho-roots-DAI6, and Ho-roots-DAI20 samples, respectively (Supplementary Table S3), with a mean coverage of 37 × per biological replicate. As compared to Ho-roots-DAI2, there were 1085 genes in Ho-roots-DAI6 that were expressed at significantly different levels (343 up-regulated, 742 down-regulated) (Fig. 4a). Between the Ho-roots-DAI2 and the Ho-roots-DAI20 samples, 1482 genes were expressed at significantly different levels (634 up-regulated, 864 down-regulated) (Fig. 4a). The total number of uniquely mapped reads for Mo-roots-DAI2 and Mo-roots-DAI6 were 115.83 and 87.76 million, respectively, with a mean coverage of 33 × per biological replicate (Supplementary Table S3). 99 significantly up-regulated genes and 172 significantly down-regulated genes were identified in the Mo-roots-DAI6-DAI2 comparison (Fig. 4a), indicating that a much lower number of DEGs in Mo-roots were detected in the DAI6-DAI2 comparison than that in the Ho-root DAI6-DAI2 comparison. Furthermore, the expression of seven housekeeping genes in Ho-roots were similar to those of Mo-roots (Supplementary Table S4), suggesting that the basic level of gene expression was consistent in rice roots infected with either *M.* or *H. oryzae*.

### Functional categorization of the DEGs

Among all DEGs, there were 90, 77, and 48 enriched GO terms for various biological processes for the Ho-roots-DAI6-DAI2, Ho-roots-DAI20-DAI6, and Mo-roots-DAI6-DAI2 comparisons, respectively (Supplementary Table S5). By comparing these three sets of enriched GO terms, we identified Ho-roots-DAI6-DAI2 specific (61 GO), Mo-roots-DAI6-DAI2 specific (19 GO), and common (29 GO) enriched terms (Supplementary Table S5). Most of the 29 commonly enriched terms were involved in responses to biotic and abiotic stresses, processes that are known to overlap. These included “response to chemical stimulus” (GO:0042221), “response to abiotic stimulus” (GO:0009628), “oxidation-reduction process” (GO:0055114), “response to other organism” (GO:0051707), and “cell wall macromolecule metabolic process” (GO:0044036). However, despite these similarities, individual genes contributing to the common enriched GO terms revealed substantial diversity. For example, 9 and 5 genes in the group “cell wall macromolecule metabolic process” were modulated in Ho-roots-DAI6-DAI2 and Mo-roots-DAI6-DAI2, respectively, but none of these genes were common in these two comparisons (Supplementary Fig. S3). Conversely, several GO terms were fungal-challenge specific (Supplementary Table S5). Most of the Mo-roots-DAI6-DAI2 specific terms were also related to responses to biotic or abiotic stress, including the terms “defense response to fungus” (GO:0050832), “chitin metabolic process” (GO:0006030), “response to water” (GO:0009415), and “siderophore biosynthetic process” (GO:0019290) (Supplementary Table S5). The term “defense response to bacterium” (GO:0042742) was enriched solely in the Ho-roots-DAI6-DAI2 comparison (Supplementary Table S5). Interestingly, most of the genes involved in responses to biotic and abiotic stresses were up-regulated in Mo-roots-DAI6-DAI2 but down-regulated in Ho-roots-DAI6-DAI2 (Supplementary Fig. S3).

One of the most striking features of the GO terms specifically enriched in the Ho-roots-DAI6-DAI2 comparison, as compared to the Mo-roots-DAI6-DAI2 comparison, was the relatively high number of GO terms involved in carbohydrate metabolism (Supplementary Table S5). This finding was consistent with the results from the metabolome analysis. These GO terms included “polysaccharide catabolic process” (GO:0000272), “cellular polysaccharide biosynthetic process” (GO:0033692), “monosaccharide catabolic process” (GO:0046365), “glycolysis” (GO:0006096), and “lignin catabolic process” (GO:0046274).

To comprehensively assess the biological functions of the DEGs, all DEGs were mapped to KEGG database terms with the goal of identifying significantly enriched metabolic or signal transduction pathways (Supplementary Table S6). Consistent with the GO enrichment analysis, pathways involved in carbohydrate metabolism, such as “starch and sucrose metabolism”, “fructose and mannose metabolism”, “glycolysis/gluconeogenesis”, and “lignin biosynthesis” were identified in the Ho-roots-DAI6-DAI2 comparison, while no obviously enriched metabolic pathways were detected in the Mo-roots-DAI6-DAI2 comparison, with the exception of “lignin biosynthesis”.

### Validation of the RNA-seq data

The expression levels of 26 genes were assessed using qRT-PCR to validate the RNA-seq results (Supplementary Fig. S4; Supplementary Table S7). Among these 26 genes, 4 genes were housekeeping genes, 14 genes were involved in the starch degradation, glycolysis, and TCA pathways, 3 genes were involved in lignin biosynthesis, and 5 genes were involved in responses to biotic or abiotic stress in the *M.* and *H. oryzae*-challenged roots. Log2 transcript fold changes (DAI6 vs. DAI2, DAI20 vs. DAI2) measured using RNA-seq and qRT-PCR were used to assess the correlations between gene expression profiles obtained using the two experimental approaches. High linear regressions were observed for the DAI6-DAI2 comparison: for *H. oryzae*, R^2^ = 0.886, and for *M. oryzae*, R^2^ = 0.860. For the qRT-PCR DAI20-DAI6 comparison, only one gene was differently expressed, which was consistent with the results of the RNA-seq analysis. Although the fold change values for the expression of some genes measured by RNA-seq or qRT-PCR were slightly different, trends of all the examined genes at the various time points were the same. These results indicate highly consistent results from both techniques. The similar consistence between the RNA-seq and qRT-PCR results were also detected for the gene expressions of *H.* and *M. oryzae* in the same RNA-seq data sets used in this study^9^, suggesting the successful application of RNA-seq to analyze the gene expressions of the interacted fungi and plants simultaneously.

### Divergent carbohydrate metabolism in rice roots in response to *H.* or *M. oryzae* colonization

**Figure 5:**
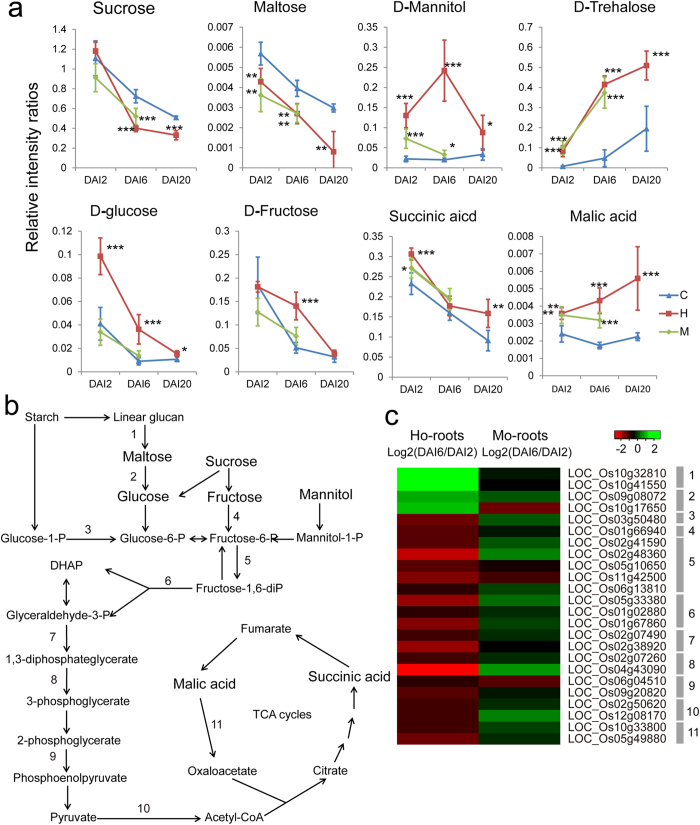
Integration of gene expression and metabolite changes for the starch degradation, glycolysis, and TCA biochemical pathways. (**a**) Changes in metabolite levels during the colonization process. DAI2, 2 days after inoculation. C, control samples; H, *H. oryzae*-challenged roots; M, *M. oryzae*-challenged roots. *P < 0.05; **P < 0.01, ***P < 0.001. (**b**) Schematic maps of the starch degradation, glycolysis, and TCA pathways. (**c**) Differential expression patterns of genes involved in the starch degradation, glycolysis and TCA pathways. Transcript fold-changes (log2) of DAI6 vs. DAI2 are indicated. 1, beta-amylase; 2, glycosyl hydrolases; 3, phosphoglucomutase; 4, fructokinase; 5, 6-phosphofructokinase; 6, aldolase; 7, glyceraldehyde-3-phosphate dehydrogenase; 8, phosphoglycerate kinase; 9, enolase; 10, dehydrogenase E; 11, malate dehydrogenase.

As compared to the Control-roots, the changes in the concentrations of most carbohydrates were similar in the Ho-roots and Mo-roots, and included reductions of sucrose and maltose and increases of mannitol and trehalose (Fig. 5a). The concentration of glucose was significantly increased in the Ho-roots as compared to the Control-roots at all stages measured. However, a similar increase of glucose was not detected in the Mo-roots. The concentration of fructose increased dramatically at DAI6 in the Ho-roots as compared to both the Control-roots-DAI6 and the Mo-roots-DAI6 samples (Fig. 5a).

The increased glucose content in the Ho-root samples is consistent with the observed decrease in maltose content, and this correlates with the observed enhanced expression of the starch hydrolyzing enzymes (Fig. 5a–c). However, a similar reduction in maltose content was detected in the Mo-root samples though the glucose concentration in the Mo-roots sample did not increase (Fig. 5a). This phenomenon can potentially be explained by the different expression patterns of genes coding for enzymes in glucose and fructose metabolism in the Ho-root and Mo-root samples. The expression of most genes encoding enzymes involved in glycolysis and the TCA cycle, including phosphoglucomutase, fructokinase, aldolase, glyceraldehyde-3-phosphate, phosphoglycerate kinase, enolase, dehydrogenase E, and malate dehydrogenase, were suppressed in the Ho-root samples, but enhanced (or not different than the controls) in the Mo-root samples (Fig. 5c). Suppressed glycolysis activity would theoretically result in the accumulation of glucose and fructose. Consistent with the suppressed expression of malate dehydrogenase in the Ho-root samples, there was an accumulation of malic acid in Ho-root samples during the colonization process; this was not detected in the Mo-root samples (Fig. 5a). These results suggest that differential gene expression programs were adopted by Ho-roots and Mo-roots to maintain different carbohydrate concentrations. These concentrations may be important in how rice roots respond differentially to the presence of a mutualist or a pathogen.

### The shikimate pathway and defensive lignification in *H.* or *M. oryzae*-challenged roots

**Figure 6:**
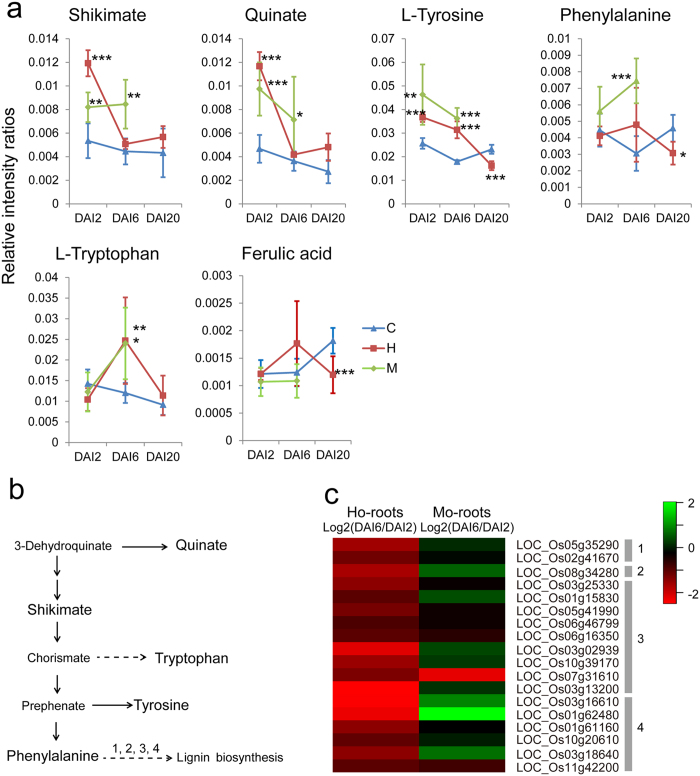
Metabolite changes in the shikimate pathway and gene expression changes in lignin biosynthesis. (**a**) Changes in metabolite levels during the colonization process. DAI2, 2 days after inoculation. C, control samples; H, *H*. *oryzae*-challenged roots; M, *M. oryzae*-challenged roots. *P < 0.05; **P < 0.01, ***P < 0.001. (**b**) Schematic maps of the shikimate pathway. (**c**) Different expression patterns of genes involved in lignin biosynthesis. Transcript fold-changes (log2) of DAI6 vs DAI2 are indicated. 1, phenylalanine ammonia-lyase; 2, cinnamoyl-CoA reductase; 3, peroxidase; 4, laccase.

Sustained increases in both quinate and shikimate were observed in the Mo-root samples, indicating apparently enhanced flux through the shikimate pathway (Fig. 6a). In contrast, the concentrations of quinate and shikimate increased at DAI2 but then decreased to normal levels at DAI6 and DAI20 in the Ho-root samples, representing a modulation of the shikimate pathway different from that observed in the Mo-root samples (Fig. 6a). Consistent with the reduced shikimate pathway activation in the Ho-root samples, the enzymes catalyzing lignin biosynthesis, including phenylalanine ammonia-lyase, cinnamoyl-CoA reductase, peroxidase, and laccase, were all down regulated at DAI6 compared to DAI2 (Fig. 6c). However, enhanced (or unchanged) expression of these genes was detected in the Mo-root samples (Fig. 6c). Phenylalanine levels increased dramatically at DAI6 in the Mo-root samples compared to the controls; no such difference was detected between the control and the Ho-root samples (Fig. 6a). Besides, as a typical phenylpropanoid metabolite, the content of ferulic acid, which involved in resistance to pathogenic fungi^24^, was decreased at DAI20 in Ho-root samples compared to the controls (Fig. 6a). These results suggested that defensive phenylpropanoid metabolism was suppressed at the onset of the endophytic interaction between *H. oryzae* and rice, but that it was enhanced or sustained in the interaction between rice and *M. oryzae*. Consistently, the elevated tryptophan contents in Ho-root samples at DAI6 decreased to the same level of controls at DAI20 (Fig. 6a), the pathway of which also involved in the defense responses of rice against pathogenic infection^25^.

## Discussion

Driven by the devastating impact of diseases in agriculture, extensive plant pathology research has been conducted that has resulted in substantial knowledge about how plants defend themselves against pathogens. There has also been intense research into how plants engage in mutualistic root symbioses^26^,^27^,^28^,^29^. In pathogenic interactions, nutrients flow from the host to pathogens; in mutalistic interactions, there is nutrient trafficking in both directions^30^. The interaction between plant roots and arbuscular mycorrhizal (AM) is a well-studied example of mutualism^31^. AM mutualism is an ancient plant-microbe interaction that is considered to have been crucial for the successful colonization of land by plants^32^,^33^. A study showed that 40% of the genes in rice roots whose expression was induced by AM were also induced when roots were infected with pathogenic fungi, revealing an ancient pattern of response to fungal infection^34^. Different from the AM symbiosis, benifical endophytism is evolutionarily transient and has originated multiple times^12^,^13^. It has been shown that some endophytes have evolved recently from pathogenic ancestors^9^. Empirical comparisons of the responses of a single host plant species to these mutualistic and pathogenic fungi with the same pathogenic ancestor can help to uncover the underling mechanism for the emergence of novel mutualistic interactions that have evolved recently.

It has been shown that *H.* and *M. oryzae* evolved from a common pathogenic ancestor^9^. Morphological observations suggested that similar root colonization strategies are used by *H.* and *M. oryzae* at the early stages of infection^9^,^23^. This assertion is bolstered here with evidence from the host plant side of the interaction showing a greater degree of divergence between the metabolite profiles of the *H.* and *M. oryzae*-treated rice roots at the middle stage (DAI6) as compared to the early stage (DAI2). Further, while the unique effects of the different fungi colonizations were evident at DAI6, there was some degree of overlap of the clusters for the Ho-root and Mo-root samples at DAI2. Even though rice roots respond similarly when challenged by these two fungi at the early stages of such interactions, our metabolic profiling analyses indicated a larger discrepancy of metabolic activity between Ho-roots and Control-roots than between Mo-roots and Control-roots at both DAI2 and DAI6, which indicates that rice roots challenged by *M.* and *H. oryzae* differentiate between day 2 and day 6 of fungal challenge. One possible explanation for this could be that the *H. oryzae*-rice interactions originate from the interaction between rice and a phytopathogenic ancestor. This more complex set of physiological responses observed in rice roots colonized by *H. oryzae* is reflected in the much larger number of DEGs that were detected in the Ho-root samples as compared to the Mo-root samples.

Many studies have revealed that sophisticated reprogramming mechanisms in a host’s metabolism and physiology are required for successful colonization and/or establishment of pathogenicity^35^,^36^,^37^. In the establishment of AM symbiosis, a consistent set of genes is expressed in plant roots, including genes involved in nutrient transport, responses to biotic and abiotic stimuli, and the regulation of developmental processes^26^,^27^,^38^,^39^. For example, transcriptional changes triggered in *Solanum lycopersicum* roots and shoots by *Glomus mosseae* were detected by microarray and were mainly involved in primary and secondary metabolism, defense, and transcriptional regulation^27^. A similar study showed that genes induced during interactions between *Medicago truncatula* and AM had functions related to sugar transport, secondary metabolism, proteolysis, and changes in signaling^26^. These results suggest that a profound molecular reprogramming occurs in the roots during AM mutualism. Most of these studies of AM mutualism are based on cDNA microarray data and are thus limited by the fact that probe sets can only be deisgned based on known transcripts; many genes may be missed^27^,^39^. To better explore the intersection between endophytic and pathogenic interaction in rice roots, we used RNA-Seq to profile transcription changes. A large number of genes displayed differential expression between the Ho-root and Mo-root samples, though few such genes were shared in common in both sample types. This result highlights the differences in the reprogramming triggered by the establishment of either a mutualistic or a pathogenic interaction. GO enrichment analysis of these DEGs identified many enriched terms, most of which related to carbohydrate metabolism and/or responses to biotic and abiotic stress. Consistently, metabolic profile analysis showed that many of the differentially accumulated metabolites were part of carbohydrate or aromatic amino acid metabolism.

It has been shown that the reinforcement of cell walls by phenolics is part of a defense reaction against both pathogens and endophytes^35^,^36^,^40^,^41^. Early defensive activation of the phenylpropanoid pathway leads to production of lignin precursors for cell wall strengthening in resistance responses^42^,^43^. Phenylpropanoids are produced from precursors derived from the shikimic acid pathway, a pathway known to be transcriptionally activated by pathogen attack^44^,^45^,^46^. In our study, a transcriptional activation of lignin biosynthetic genes accompanied by elevated phenyalanine and tyrosine accumulation levels was evident in the Mo-roots. Consistently, the content of shikimate, a metabolite upstream of phenylalanine, was also observed to increase. Activation of the shikimate pathway leads to the biosynthesis of the lignin precursor compounds that are used for cell-wall strengthening in resistance responses^43^. The enhancement of the shikimate pathway and the transcriptional activation of lignin biosynthesis genes observed here indicate the presence of induced defenses in rice in response to *M. oryzae*. This has also been reported in other host-pathogen interactions^35^,^36^. Similarly, the shikimate pathway was induced in the Ho-root samples at DAI2, indicating induced plant defense responses in response to *H. oryzae*. However, at DAI6 and DAI20, the activities of shikimate pathway and lignin biosynthesis in the Ho-root samples were at the same levels as in the control samples. Similarly, defense- and stress-response genes were also induced in *M. truncatula* roots during AM mutualism^38^. Different from what occurs in AM mutualism, the rice defense responses observed in our study were activated but subsequently suppressed in the endophytic interaction with *H. oryzae*. DEGs involved in responses to biotic and abiotic stress were up-regulated in the Mo-root samples but down-regulated in the Ho-root samples. These results reveal the distinct responses of rice to infection by different fungi; showing that the defense responses in the interaction of rice with *H. oryzae* are suppressed, unlike the situation with rice and *M. oryzae*. These observations are consistent with the knowledge that the *H. oryzae* genome encodes more elements to suppress plant defense responses than does the *M. oryzae* genome^9^, including LysM-bearing proteins that are secreted and interact with chitin and a larger number of small secreted cysteine-rich proteins (SSCRP). Further, it is known that some virulence-associated gene orthologs are down regulated in *H. oryzae* but up regulated in *M. oryzae*; none of the SSCRP orthologs in *H.* and *M. oryzae* were regulated identically between the two species^9^. These different gene expression patterns in *H. oryzae* are also related to the suppression of plant defense responses.

It is known that in susceptible host-pathogen interactions in plants, invertase activity increases after infection^21^,^35^,^47^. This is accompanied by a progressive accumulation of hexoses and sucrose, and a reduction in photosynthesis in host leaves^47^. For example, in *Brachypodium distachyon* infected with *M. oryzae*, a rapid elevation of sucrose, glucose, and fructose content in leaves is evident when visible lesions first appear, and this is mirrored by a large increase in mannitol content^21^. Glucose and fructose levels in the rice roots of this study were not significantly affected by *M. oryzae*, though the levels of sucrose and maltose decreased, a result that may reflect the tissue-adapted invasion strategy of *M. oryzae*^22^. Unlike pathogens, mutualistic partners can improve the growth and fitness of the host plant by ‘paying’ their hosts with nutrients that are difficult to access by the plant or through indirect ‘payment’ such as the production of plant protective anti-herbivory alkaloids^48^. Improved plant fitness as a result of this interaction, however, has a price. For example, up to 20% of photosynthetically fixed carbon of host plants is translocated to the AM fungi which are obligate symbionts that entirely depend on carbon and energy supplied to them by their autotrophic hosts^49^,^50^. Together with their fungal partner, the root systems of ectomycorrhizal plants can receive about half of the plant’s photosynthetically fixed carbon^51^. In contrast to the Mo-root samples, the glucose and fructose levels increased dramatically in the Ho-root samples. For AM, carbon produced by the host plant is taken up in the form of glucose or hexose^52^,^53^. The increase in free hexose content found in the Ho-root samples is typical for a sink tissue, and the free hexoses within rice roots could be used by *H. oryzae* as an easily accessible carbon source. In addition to its role as a carbon source, hexose has been shown to play other important roles in plant-fungi interactions, such as inhibiting the fungal expression of plant cell wall degrading enzymes^9^. It has been shown that the hexose content in rice is correlated to the nutritional preferences of *H.* or *M. oryzae*, and play important roles in the evolution of the interactions between rice and *H.* or *M. oryzae* leading to either mutualism or disease. However, the cause of the different contents of glucose and fructose in rice roots with different fungi-plant interactions is still unclear. The metabolite profiling and comparative transcriptomics analysis in this study imply that the accumulated glucose and fructose content in the Ho-root samples may relate to the apparently suppressed flux of glucose through glycolysis and the TCA cycle that we deduced from the suppressed expression of genes encoding enzymes involved in glycolysis and the TCA cycle. Sucrose degradation can also contribute to pools of fructose, the precursor of fructose phosphate. With the suppressed expression of fructokinase in the Ho-root samples, less fructose would theoretically be available for glycolysis, which could lead to an increase in fructose content. The rapid increase of glucose and fructose in *H. oryzae*-challenged roots was accompanied by a very significant increase in mannitol, especially at DAI6. Mannitol has been previously proposed to be an important carbohydrate for fungal growth^54^. In the interactions between fungal *Neotyphodiumlolii* endophytes and *Lolium perenne*, it has been shown that mannitol levels are significantly positively correlated with fungal biomass^36^. As mannitol is membrane-impermeable, the conversion of imported hexoses to mannitol might maintain a gradient for the continued uptake and sequestration of carbohydrates^21^.

It is now clear that clavicipitaceous endophytes arose from insect-parasitic ancestors^14^. However, this evolutionary pattern does not fit the often-expected transition from phytopathogens to endophytes^6^. We addressed this transition by suggesting a novel evolutionary model of the beneficial endophyte *H. oryzae*, which originated from a phytopathogenic ancestor^9^. Although *M. oryzae* preferentially infects aerial parts of rice with appressoria, Tucker^55^ proposed that hyphopodia represent primitive appressoria and that the ability to develop appressorium-mediated penetration is acquired later with the incorporation of novel genes or functions involved in surface recognition (*pth11* and *mpg1*), peg formation (*gas1*, *gas2* and *pls1*), turgor formation (*cpkA*) and melanin synthesis. *M. poae* and *G. graminis*, the closely related fungi of *H.* and *M. oryzae*, are both pathogenic and obligated root-infection^9^. Similar to *M. oryzae*, both *M. poae* and *G. graminis* share the same pathogenic ancestor with *H. oryzae*^9^. Thus, the root-infection by these fungi is a more ancient character than the aerial part-infection, which is preserved by both *H. oryzae* and its closely related pathogenic fungi, indicating that the comparative analysis of *M.* and *H. oryzae* on rice roots can illuminate well the novel co-evolution pattern of fungi-host interaction. In the present study, our findings highlight the importance of the host in the evolutionary processes of fungi. The development of these diverse interaction strategies may have been caused by competition between *H. oryzae* and related species such as *M. oryzae* to occupy different ecological niches. It has been shown that *H. oryzae* colonization can restrict root infection by *M. oryzae*^23^, suggesting that competition between these closely related species does indeed occur. To make full use of a given carbon source, the ecological niches for their common pathogenic ancestors might have changed. This is revealed by the fact that *H. oryzae* has lost the ability to infect the aerial parts of host plant, a capacity that is retained by *M. oryzae*^9^. We have developed an experimental system that allows us to use the same plant to study the colonization biology of two different types of plant-fungi interactions with closely related fungi. Our results with this experimental system contribute significantly to the knowledge and genomics resources of the study of plant-fungi interactions and should prove useful in attempts to employ friendly microbes in crop production systems to control devastating plant diseases.

## Methods

### Biological materials and culture conditions

The fungal endophyte *H. oryzae* strain R5-6-1, the rice blast fungus *M. oryzae* strain Guy11, and the blast-susceptible rice cultivar CO-39 (*O. sativa*) were used in these experiments. Rice seeds were surface sterilized as described previously^23^. To promote root growth, the sterilized seeds were planted in half-strength Murashige & Skoog (1/2 MS) solid medium and cultured in the dark at 30 °C for 4 days^22^. The seedlings were then transferred to sterilized square culture dishes (13 × 13 cm), with 5 plants per plate, and grown vertically for an additional 6 days with a 16-h light/8-h dark photoperiod at 28/24 °C. *H. oryzae* strain R5-6-1 was cultured on complete medium (CM)^56^ at 25 °C in the dark; *M. oryzae* strain Guy11 was cultured on the same medium, but with a 16-h light/8-h dark photoperiod.

### Inoculation, co-culture, and harvest of rice roots

Conidia of *M. oryzae* were harvested from 10-day-old cultures grown on solid CM. Germinating phialidic conidia of *H. oryzae* were harvested from 4-day-old potato dextrose broth (with 5 g glucose/L), as conidia harvested from cultures grown on solid CM are not able to germinate^9^. Inoculations and co-culturing were performed as described previously^9^. Based on a previous study^9^, we chose to sample rice roots infected by *H. oryzae* at 2, 6, and 20 days after inoculation (DAI) and to sample rice roots infected by *M. oryzae* at 2 and 6 DAI (at DAI20, most of the roots infected by *M. oryzae* were dead). These particular sample tissues and time points represent contexts during which the main differences of the respective colonization strategies employed by *H. and M. oryzae* should be detectable. All samples were harvested and immediately flash frozen in liquid nitrogen. The control samples were prepared using sterile water instead of a conidia suspension for inoculation.

### Sample Preparation for GC-MS analysis

Grouped roots from 10 independent rice plants were grouped as a single experimental replicate, and 5-6 such biological replicates were prepared and analyzed for each treatment. Briefly, the root samples (10 mg dry weight per sample) were freeze-dried for 48 h and ground to a powder. Quality control (QC) samples were obtained by thoroughly blending the same amount of each ground sample^57^. The sample preparation for the GC-MS analysis was performed as previously described^9^. Chromatography was performed on an Agilent 7890/5975C-GC-MSD using an Agilent DB-5MS UI column (30 m × 0.25 mm × 0.25 mm) and helium as a carrier gas.

### Metabolite assays by GC-MS

Samples were analyzed in random order with QC samples inserted every eight samples in the data acquisition sequence. For each sample analysis, 1 μL of derivatized sample was injected with a split ratio of 10:1. The parameters and the procedures for the GC-MS analysis were performed as previously described^9^. Components of the total ion chromatogram were extracted using the Automatic Mass Spectral De-convolution and Identification System (AMDIS) (NIST, Gaithersburg, MD, USA). Metabolites were identified by comparing their mass spectra with those of commercial standards^57^,^58^,^59^. The peak areas were analyzed with an in-house automatic integration method established in Agilent MSD ChemStation. Manual corrections were performed to guarantee the accuracy of the integrations. The relative intensities of the various metabolites were obtained by normalizing the intensity of individual ion traces, which are indicative of the respective compounds, to the response of an internal reference compound. The internal standard and any known artificial peaks were removed from the results matrix^57^.

### Statistical and multivariate analysis

The data matrix was imported into SIMCA-P 13.0 (Umetrics, Umeå, Sweden) for multivariate data analysis, and Pareto-scaled to minimize the influence of baseline deviations and noise^60^. Principal component analysis (PCA) was used initially to obtain an understanding of the relationships among the samples and metabolites. An orthogonal partial least-squares discriminate analysis (OPLS-DA) was performed to further differentiate the contributions of particular metabolites to the separations of the different sample groups. The efficiency and reliability of the OPLS-DA models were verified by percent variation of the x and y variables explained by the model (R2X, R2Y) and the predictive performance of the model (Q2)^57^. In addition, permutation tests were performed to validate the OPLS-DA models^61^.

### Transcriptome analysis

The technique of RNA-seq was used in this study for the advantages of analyzing the gene expressions of the interacted fungi and plants simultaneously without the indiscrimination. The RNA-seq data of Mo-roots and Ho-roots used in the transcriptome analysis were same to the data sets used in our previous report^9^ which only focused on the fungi in the rice-fungi interaction. As the genome sequences of rice cultivar CO-39, *M.* and *H. oryzae* are available^9^,^62^,^63^, all the clean reads were mapped to the genome sequences using TopHat v 2.0.9^64^, and an expression profile was created using Cufflinks v2.0.2^65^. The abundances of gene expression were reported as normalized fragments per kb of transcript per million mapped reads. Transcripts with a significant p-value (<0.05) and a greater than two-fold change in transcript level between the Ho-roots and Mo-roots were considered as ‘differentially expressed’. All the p-values were corrected for false discoveries resulting from multiple hypothesis testing using the Benjamini-Hochberg procedure. GO enrichment analysis was performed using a hypergeometric test with TopGO^66^, and p-values were adjusted with Bonferroni correction for multiple testing. The FDR criterion was selected as FDR <0.05. Heatmaps of gene expression profiles were generated using R (www.R-project.org) based on significant expression changes (log2 fold change).

### Quantitative RT-PCR

Total RNA of Ho-roots or Mo-roots was extracted as described above. Three independent biological replicates were performed for each sample. Quantitative RT-PCR was performed as described previously^9^. Four commonly used house-keeping genes were used (Supplementary Table S7). One of the house-keeping genes encoding elongation factor 1-alpha was used as an endogenous control, and the stability of expression of the three other house-keeping genes was assessed. The relative gene expression levels of target genes were calculated using the 2^–∆∆Ct^ method^67^. The sequences of the primers used in the qRT-PCR analysis are listed in Supplementary Table S7.

## Additional Information

**How to cite this article**: Xu, X.-H. *et al.* Friend or foe: differential responses of rice to invasion by mutualistic or pathogenic fungi revealed by RNAseq and metabolite profiling. *Sci. Rep.* **5**, 13624; doi: 10.1038/srep13624 (2015).

## Supplementary Material

Supplementary Information

Supplementary Information
