# Supplementary material for: Friend or foe: differential responses of rice to invasion by mutualistic or pathogenic fungi revealed by RNAseq and metabolite profiling
Source: Sci Rep. 2015 Sep 8;5:13624. doi: 10.1038/srep13624 (PMC4642567; doi:10.1038/srep13624)
Supplement: Supplementary Information [file srep13624-s1.pdf]

Supplementary information

Friend or foe: differential responses of rice to invasion by mutualistic or pathogenic fungi revealed by RNAseq and metabolite profiling

Xi-Hui Xu[1], Chen Wang[1], Shu-Xian Li[1], Zhen-Zhu Su[1], Hui-Na Zhou[2], Li-Juan Mao[1], Xiao-Xiao Feng[1], Ping-Ping Liu[2], Xia Chen[2], John Hugh Snyder[2], Christian P. Kubicek[3], Chu-Long Zhang[1*], Fu-Cheng Lin[1,2*]

[1] State Key Laboratory of Rice Biology, Institute of Biotechnology, Zhejiang University, Hangzhou, 310058, China

[2] Zhengzhou Tobacco Research Institute of CNTC, Zhengzhou 450001, China

[3] Austrian Center of Industrial Biotechnology (ACIB), c/o Vienna University of Technology, 1060 Vienna, Austria.

*Corresponding authors: e-mail: clzhang@zju.edu.cn or fuchenglin@zju.edu.cn;

Tel: 0086-571-88982291 or 0086-571-88982183;

Fax: 0086-571-88982291

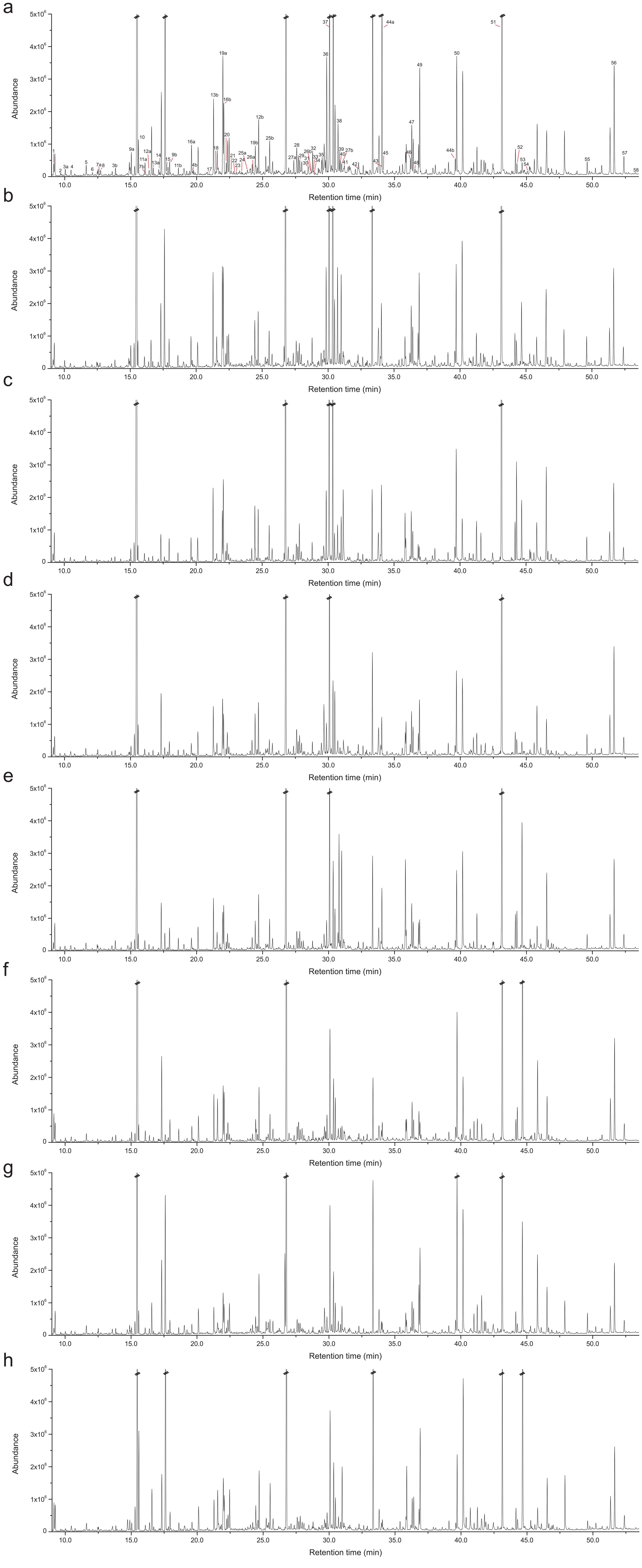

Supplementary Figure S1 Representative GC-MS total ion chromatograms of rice roots. (a) Control-roots-DAI2. (b) Ho-roots-DAI2. (c) Mo-roots-DAI2. (d) Control-roots-DAI6. (e) Ho-roots-DAI6. (f) Mo-roots-DAI6. (g) Control-roots-DAI20. (h) Ho-roots-DAI20. The labeled peaks are listed in Supplementary Table 1. Control-roots-DAI2, Control-roots-DAI6, and Control-roots-DAI20 refer to control roots at 2, 6, and 20 days after inoculation, respectively. Ho-roots-DAI2, Ho-roots-DAI6, and Ho-roots-DAI20 refer the *H. oryzae*-challenged roots at 2, 6, and 20 days after inoculation, respectively. Mo-roots-DAI2 and Mo-roots-DAI6 refer to the *M. oryzae*-challenged roots at 2 and 6 days after inoculation, respectively.

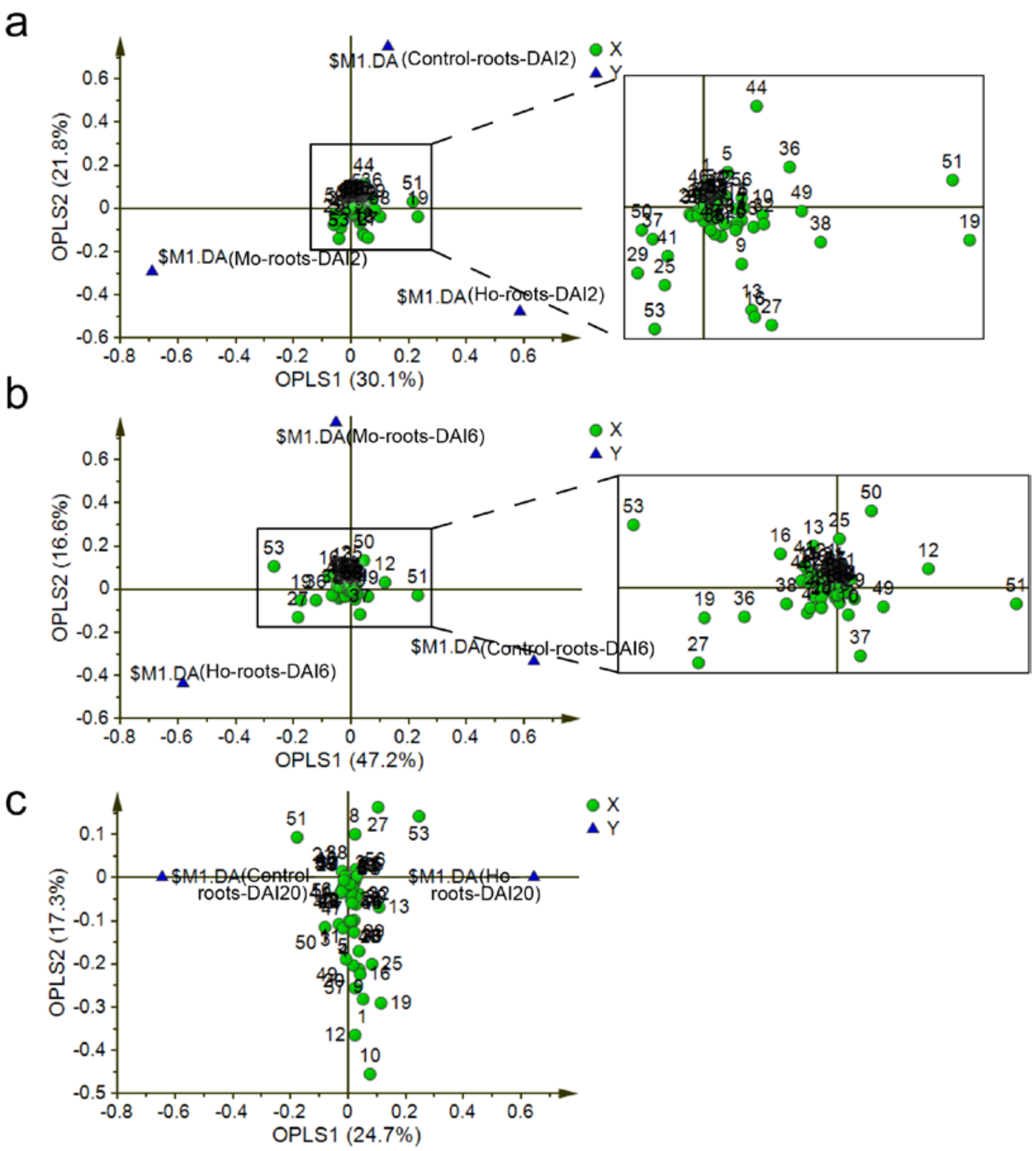

Supplementary Figure S2 Loading plots of OPLS-DA models based on metabolite profiling data of rice roots infected with *H. oryzae* (Ho-roots), *M. oryzae* (Mo-roots) or sterile water (Control-roots) at 2 (a), 6 (b) and 20 (c) days after inoculation (DAI), respectively. In the loading plot, the closer to the origin, the smaller contribution a metabolite makes to the discrimination. Each point represents a metaboliteand the numbers above the points indicate the metabolites in Supplementary Table S1.

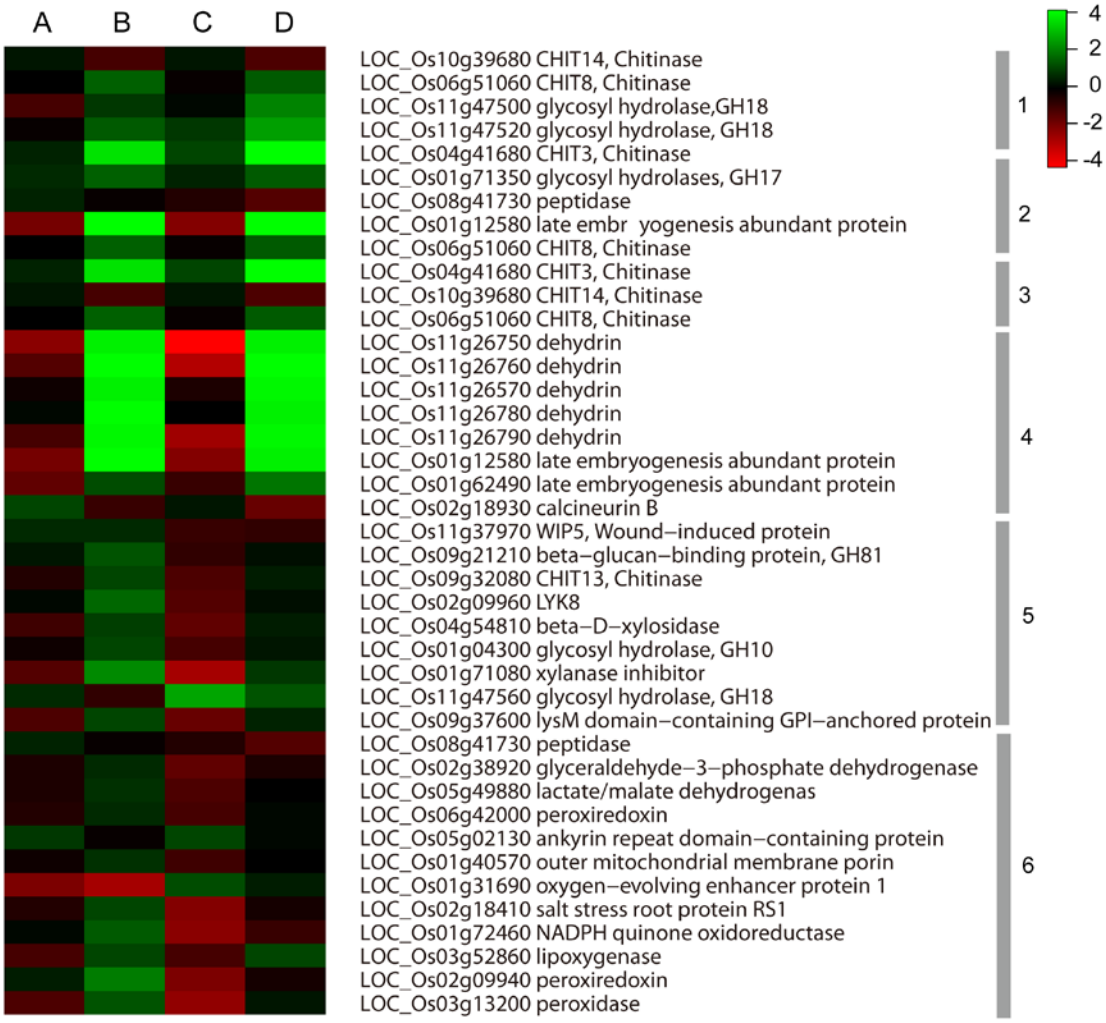

Supplementary Figure S3 Diverse expression patterns of genes responding to biotic or abiotic stress in the *M. and H. oryzae*-challenged roots. Log2 transcript fold changes were measured A, Log2(Mo-roots-DAI2/Ho-roots-DAI2); B, Log2(Mo-roots-DAI6/Ho-roots-DAI6), C, Log2(Ho-roots-DAI6/Ho-roots-DAI2); D, Log2(Mo-roots-DAI6/Mo-roots-DAI2). 1 and 2, cell wall macromolecule metabolic process (GO:0044036, *M and H. oryzae*-challenged roots, respectively); 3, defense response to fungus (GO:0050832); 4, chitin metabolic process (GO:0006030); 5, defense response to bacterium (GO:0042742); 6, response to water (GO:0009415).

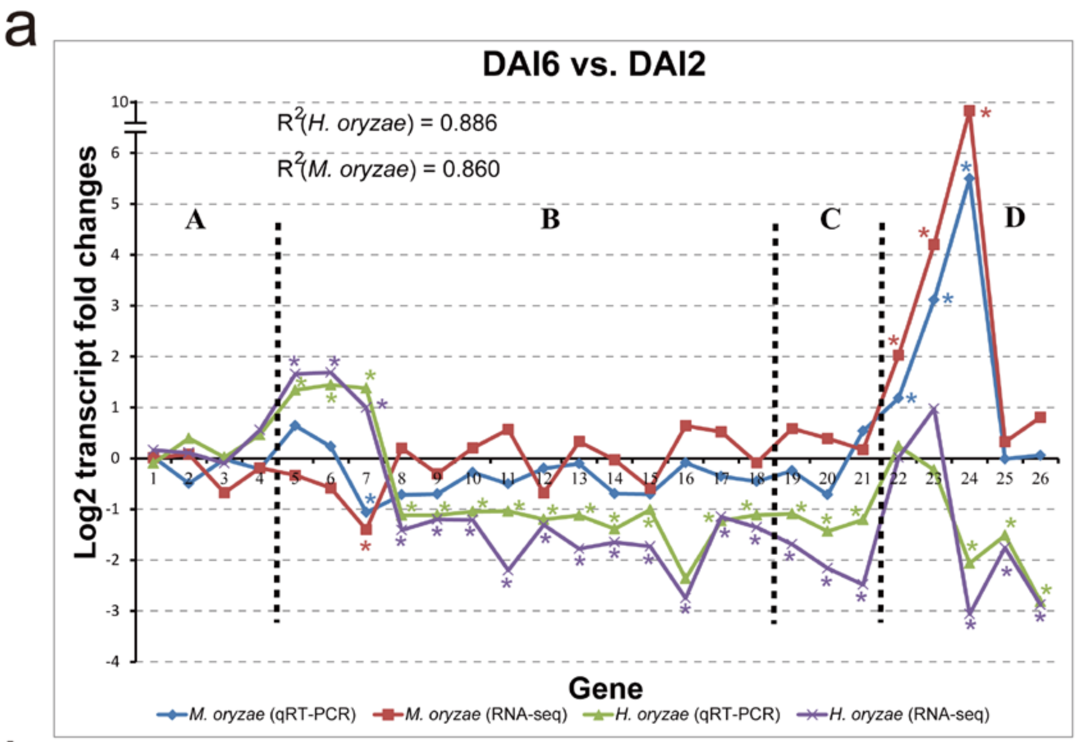

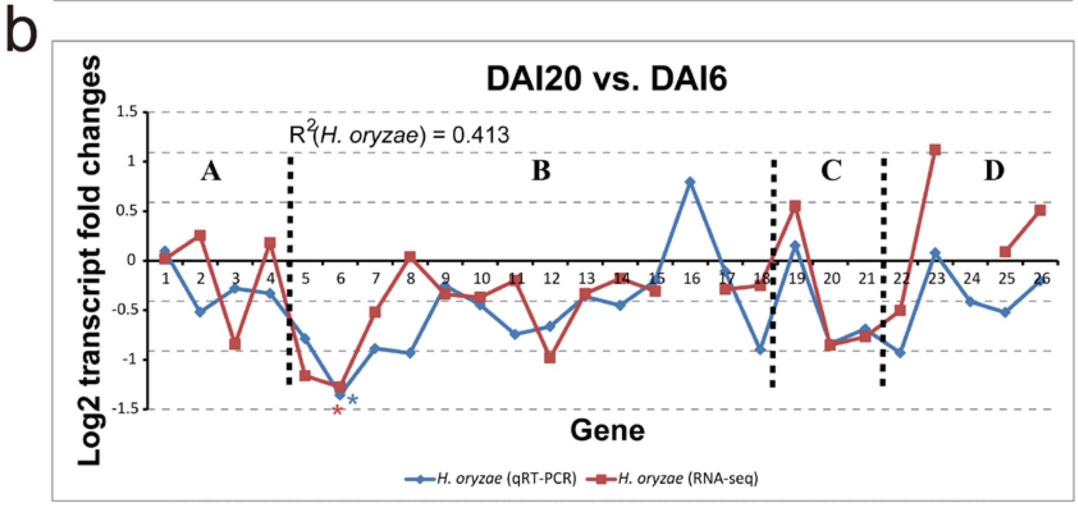

Supplementary Figure S4 Verification of the RNA-seq data using quantitative RT-PCR. (a) Transcript fold-changes of DAI6 vs. DAI2 for both *H. oryzae* and *M. oryzae*. (b) Transcript fold-changes of DAI20 vs. DAI2 for *H. oryzae*. A, housekeeping genes; B, genes involved in the starch degradation, glycolysis and TCA pathways; C, genes involved in lignin biosynthesis; D, diversely expressed genes responding to biotic or abiotic stress in the *M.* and *H. oryzae*-challenged roots. Asterisks indicate significant differences of DAI6 when compared to DAI2 or DAI20 to DAI6. All the genes used are listed in Supplementary Table S7.

Supplementary Table S1. Primary and secondary metabolites identified by GC-MS from all the samples.

Primary Metabolites[1]				Secondary Metabolites
**Amino Acid**	**Sugars and polyols**	**Organic and fatty acids**	**Others**	
L-Valine, 3a	Glycerol, 10a	Propanoic acid, 1b	Phosphoric acid, 8b	Oleanitrile, 43b
L-Alanine, 4a	D-Mannitol, 27b	Glycolic acid, 2b	Niacinamide, 17b	Oleamide, 50b
L-Isoleucine, 6a	Glycerol 1-phosphate, 28b	Ethanedioic acid, 5b	Allantoin, 37b	Stigmasterol, 56a
L-Proline, 7a	D-Fructose, 36a	Succinic acid, 13a	2-Pyrrolidone-5-carboxylic acid, 18b	beta-Sitosterol, 57a
Serine, 9a	D-Glucose, 38a	Glyceric acid, 14b	adenosine-5-monophosphate , 55b	
L-Threonine, 11a	Inositol, 44a	Malic acid, 15a		
Glycine, 12a	Sucrose, 51a	Butanoic acid, 20b		
L- Aspartate, 16b	Lactose, 52a	L-Threonic acid, 23b		
L- Glutamate, 19b	D-Trehalose, 53a	α-Ketoglutaric acid, 24b		
Phenylalanine, 21a	Maltose, 54a	3-phosphoglyceric acid, 30b		
L-Cysteine, 22a	Raffinose, 58a	Shikimate, 31a		
L-Asparagine, 25b		Citric acid, 32b		
Ornithine, 26b		Isocitric acid, 33b		
L-Glutamine, 29b		Quiniate, 35a		
Citrulline, 34b		Ferulic acid, 45b		
L-Histidine, 39a		Linoleic acid, 47a		
L-Lysine, 40a		trans-Octadecenoic acid, 48b		
L-Tyrosine, 41a		Stearic acid, 49a		
L-Tryptophan, 46a		Pantothenic acid, 42b		

[1]Identification was performed by comparing the mass spectra and retention index with those of commercial standards (a) and comparison with published database (b)

Supplementary Table S2 . The abundances of metabolites identified by GC-MS. The IDs are listed in Supplementary Table 1

ID	Control-roots-DAI2	Ho-roots-DAI2	Mo-roots-DAI2	Control -roots-DAI2	Ho-roots-DAI6	Mo-roots-DAI6	Control -roots-DAI20	Ho-roots-DAI20
1	0.02648±0.00484	0.02161±0.00462	0.02223±0.00770	0.01874±0.00330	0.01395±0.00298	0.02350±0.01665	0.01308±0.00272	0.01520±0.00124
2	0.01001±0.00166	0.00953±0.00210	0.00711±0.00089	0.00777±0.00112	0.00642±0.00069	0.00635±0.00081	0.01090±0.00172	0.01095±0.00210
3	0.05834±0.00719	0.07025±0.00325	0.05315±0.00741	0.04289±0.01480	0.05476±0.00661	0.05543±0.00936	0.04019±0.00320	0.03294±0.00591
4	0.03084±0.00365	0.04167±0.00408	0.03405±0.01122	0.02792±0.00193	0.04176±0.00456	0.02497±0.00673	0.01965±0.00565	0.03135±0.01323
5	0.02160±0.00289	0.01523±0.00091	0.01007±0.00626	0.01586±0.00248	0.01384±0.00107	0.01550±0.00740	0.02418±0.00369	0.02053±0.00694
6	0.01217±0.00172	0.01449±0.00118	0.00836±0.00130	0.00768±0.00252	0.01120±0.00120	0.00874±0.00223	0.01010±0.00233	0.00689±0.00165
7	0.01617±0.00185	0.02269±0.00244	0.01375±0.00464	0.01245±0.00390	0.02428±0.00634	0.00891±0.00268	0.00684±0.00076	0.01168±0.00299
8	0.00283±0.00097	0.00465±0.00244	0.00355±0.00115	0.00311±0.00179	0.00335±0.00153	0.00412±0.00263	0.00101±0.00054	0.00765±0.01226
9	0.08690±0.01117	0.11857±0.01448	0.09679±0.01452	0.06201±0.01189	0.07633±0.00989	0.07517±0.00919	0.05471±0.00190	0.07105±0.02008
10	0.04205±0.00612	0.04526±0.00639	0.03514±0.00503	0.04519±0.00692	0.04361±0.01054	0.03136±0.00941	0.04472±0.01326	0.10839±0.09913
11	0.02136±0.00188	0.02617±0.00213	0.01991±0.00211	0.01645±0.00425	0.02611±0.00390	0.02778±0.00540	0.01611±0.00094	0.01690±0.00414
12	0.18700±0.00975	0.19682±0.00748	0.20750±0.01679	0.19199±0.00372	0.10187±0.10738	0.20245±0.02574	0.21015±0.03751	0.22568±0.04759
13	0.23263±0.02684	0.30638±0.01492	0.27127±0.02431	0.15973±0.01822	0.17706±0.02466	0.19426±0.02650	0.09096±0.02545	0.15859±0.03480
14	0.00769±0.00105	0.00940±0.00080	0.00534±0.00065	0.00595±0.00077	0.00646±0.00057	0.00448±0.00094	0.00403±0.00143	0.00416±0.00093
15	0.00241±0.00047	0.00358±0.00034	0.00348±0.00048	0.00174±0.00019	0.00431±0.00073	0.00319±0.00045	0.00225±0.00022	0.00559±0.00182

16	0.23515±0.01587	0.31687±0.02987	0.27210±0.03734	0.10951±0.01685	0.16407±0.01418	0.17199±0.01384	0.10648±0.00650	0.12048±0.02724
17	0.00079±0.00020	0.00169±0.00018	0.00120±0.00031	0.00074±0.00013	0.00109±0.00028	0.00085±0.00013	0.00074±0.00004	0.00076±0.00010
18	0.01180±0.00108	0.01735±0.00137	0.01166±0.00289	0.01135±0.00169	0.01181±0.00212	0.00669±0.00115	0.00706±0.00235	0.00866±0.00087
19	0.61899±0.06034	0.74366±0.07109	0.48998±0.08747	0.37172±0.04511	0.54692±0.06636	0.42686±0.06556	0.26973±0.07044	0.36486±0.05023
20	0.03528±0.00680	0.04591±0.00488	0.03625±0.00843	0.02721±0.00688	0.03588±0.00852	0.02446±0.00558	0.01980±0.00690	0.02659±0.02115
21	0.00446±0.00100	0.00412±0.00057	0.00562±0.00148	0.00305±0.00105	0.00479±0.00224	0.00745±0.00136	0.00458±0.00081	0.00307±0.00069
22	0.00152±0.00081	0.00156±0.00065	0.00200±0.00081	0.00145±0.00051	0.00149±0.00025	0.00143±0.00058	0.00117±0.00026	0.00128±0.00034
23	0.00173±0.00012	0.00207±0.00014	0.00177±0.00030	0.00154±0.00022	0.00144±0.00034	0.00139±0.00027	0.00126±0.00016	0.00097±0.00013
24	0.00169±0.00075	0.00233±0.00079	0.00178±0.00081	0.00202±0.00044	0.00184±0.00031	0.00148±0.00070	0.00127±0.00030	0.00169±0.00022
25	0.03213±0.01749	0.06650±0.01256	0.07664±0.02190	0.03910±0.01877	0.03851±0.00629	0.07232±0.03310	0.02108±0.00922	0.06388±0.02741
26	0.00376±0.00094	0.00596±0.00052	0.00497±0.00092	0.00309±0.00048	0.00714±0.00287	0.00758±0.00223	0.00777±0.00120	0.01220±0.00677
27	0.02213±0.00700	0.13001±0.03012	0.07254±0.02408	0.02011±0.00390	0.24177±0.07594	0.03245±0.01123	0.03344±0.01437	0.08756±0.04323
28	0.00455±0.00075	0.00807±0.00129	0.00507±0.00106	0.00399±0.00077	0.00937±0.00283	0.00473±0.00110	0.00373±0.00019	0.00501±0.00175
29	0.02552±0.00756	0.04928±0.00955	0.07148±0.02643	0.03627±0.00513	0.03080±0.01228	0.01998±0.00845	0.01909±0.00277	0.02237±0.00883
30	0.00069±0.00018	0.00102±0.00025	0.00123±0.00025	0.00045±0.00019	0.00036±0.00010	0.00064±0.00017	0.00027±0.00005	0.00024±0.00003
31	0.00535±0.00148	0.01192±0.00111	0.00819±0.00125	0.00445±0.00111	0.00507±0.00043	0.00845±0.00207	0.00432±0.00206	0.00567±0.00092
32	0.03367±0.00592	0.04495±0.01190	0.02798±0.00702	0.01543±0.00406	0.01505±0.00206	0.01285±0.00217	0.00650±0.00397	0.01454±0.00303

33	0.00088±0.00017	0.00144±0.00030	0.00083±0.00011	0.00061±0.00008	0.00054±0.00009	0.00058±0.00008	0.00041±0.00004	0.00069±0.00011
34	0.00069±0.00015	0.00073±0.00010	0.00060±0.00007	0.00062±0.00007	0.00120±0.00033	0.00121±0.00029	0.00070±0.00006	0.00127±0.00051
35	0.00467±0.00117	0.01167±0.00120	0.00973±0.00225	0.00363±0.00082	0.00418±0.00010	0.00713±0.00365	0.00273±0.00099	0.00481±0.00116
36	0.18448±0.06001	0.18125±0.01135	0.12750±0.02945	0.05121±0.01121	0.14037±0.02955	0.07588±0.01868	0.03190±0.01156	0.03783±0.00826
37	0.16959±0.03425	0.18140±0.00638	0.19686±0.04870	0.18764±0.01989	0.17344±0.01981	0.10902±0.02488	0.10838±0.01777	0.11967±0.02323
38	0.04096±0.01397	0.09858±0.01566	0.03388±0.01119	0.00900±0.00307	0.03624±0.01253	0.01365±0.00438	0.01071±0.00188	0.01533±0.00267
39	0.00293±0.00049	0.00350±0.00071	0.00418±0.00149	0.00378±0.00073	0.00523±0.00176	0.00447±0.00247	0.00253±0.00026	0.00666±0.00202
40	0.00645±0.00105	0.00646±0.00080	0.00522±0.00036	0.00405±0.00060	0.00830±0.00180	0.00967±0.00242	0.00533±0.00105	0.00520±0.00111
41	0.02564±0.00224	0.03670±0.00193	0.04629±0.01279	0.01788±0.00063	0.03138±0.00362	0.03620±0.00448	0.02311±0.00189	0.01618±0.00188
42	0.00376±0.00056	0.00318±0.00067	0.00205±0.00031	0.00289±0.00020	0.00219±0.00056	0.00221±0.00027	0.00191±0.00051	0.00152±0.00014
43	0.00620±0.00262	0.00693±0.00144	0.00528±0.00266	0.00744±0.00063	0.01092±0.00527	0.00650±0.00292	0.00933±0.00305	0.00714±0.00090
44	0.22150±0.01518	0.17196±0.03663	0.13916±0.03890	0.02734±0.00950	0.03387±0.01052	0.02252±0.00554	0.01492±0.00462	0.01815±0.00238
45	0.00121±0.00025	0.00121±0.00011	0.00107±0.00026	0.00124±0.00025	0.00177±0.00077	0.00108±0.00031	0.00182±0.00023	0.00120±0.00034
46	0.01425±0.00345	0.01041±0.00276	0.01224±0.00473	0.01201±0.00243	0.02468±0.01047	0.02400±0.00867	0.00913±0.00246	0.01139±0.00480
47	0.01398±0.00319	0.01863±0.00211	0.01721±0.00380	0.01165±0.00191	0.01881±0.00500	0.01778±0.00418	0.00788±0.00198	0.00940±0.00280
48	0.00337±0.00065	0.00321±0.00086	0.00321±0.00044	0.00278±0.00027	0.00340±0.00095	0.00370±0.00185	0.00283±0.00018	0.00278±0.00042
49	0.09594±0.06741	0.12349±0.07537	0.06024±0.05373	0.12572±0.07979	0.08986±0.05245	0.07733±0.07589	0.12051±0.08823	0.10886±0.09526

50	0.11159±0.01182	0.11160±0.00698	0.12655±0.02664	0.12930±0.02463	0.08773±0.02755	0.13748±0.02858	0.16091±0.06395	0.10887±0.01235
51	1.10748±0.17630	1.18112±0.08810	0.91144±0.14106	0.72422±0.06640	0.40080±0.03466	0.51965±0.08214	0.50735±0.01547	0.32996±0.04688
52	0.02955±0.00614	0.11418±0.03957	0.31772±0.07555	0.02769±0.00879	0.02952±0.00921	0.06270±0.02651	0.03950±0.01976	0.06796±0.03994
53	0.00751±0.00223	0.08272±0.02714	0.10054±0.03403	0.04796±0.04185	0.41512±0.04274	0.37383±0.07728	0.19473±0.11207	0.50908±0.07178
54	0.00568±0.00057	0.00428±0.00067	0.00361±0.00082	0.00395±0.00040	0.00270±0.00050	0.00271±0.00045	0.00298±0.00020	0.00015±0.00004
55	0.00039±0.00016	0.00074±0.00018	0.00096±0.00029	0.00035±0.00015	0.00042±0.00007	0.00070±0.00029	0.00027±0.00005	0.00235±0.00240
56	0.08184±0.00525	0.07951±0.00636	0.07172±0.00811	0.08777±0.00265	0.08618±0.00878	0.08417±0.00653	0.08537±0.01073	0.09476±0.00614
57	0.00509±0.00072	0.00475±0.00099	0.00510±0.00095	0.00498±0.00032	0.00473±0.00069	0.00674±0.00167	0.00437±0.00029	0.00484±0.00064
58	0.00135±0.00040	0.00142±0.00017	0.00072±0.00022	0.00125±0.00079	0.00063±0.00030	0.00025±0.00004	0.00152±0.00070	0.00038±0.00004

Supplementary Table S3. Amount of reads mapped to plant and fungal genomes.

	**Ho-roots -DAI2[1]**	**Ho-roots -DAI6**	**Ho-roots -DAI20**	**Mo-roots-DAI2[2]**	**Mo-roots-DAI6**
Repetition 1					
Reads unique mapped to Rice (Mb)	32.01	28.78	36.59	40.7	26.94
Reads unique mapped to Fungi (Mb)	0.84	1.11	2.24	2.64	10.07
Repetition 2					
Reads unique mapped to Rice (Mb)	45.7	36.38	36.2	38	32.91
Reads unique mapped to Fungi (Mb)	1.02	2.03	3.36	4.32	9.83
Repetition 3					
Reads unique mapped to Rice (Mb)	44.01	44.65	30.14	37.12	28.72
Reads unique mapped to Fungi (Mb)	1.46	3.38	4.73	1.66	8.93

[1]Ho-roots-DAI2, Ho-roots-DAI6, Ho-roots-DAI20 refer to transcripts expressed of *H. oryzae*-challenged roots at 2, 6 and 20 days after inoculation, respectively.
[2]Mo-roots-DAI2 and Mo-roots-DAI6 refer to transcripts expressed by *H. oryzae*-challenged roots at 2 and 6 days after inoculation.

Supplementary Table S4. Expressions of seven housekeeping genes in *H.* and *M. oryzae*-chanllenged rice roots.

Gene	Gene product	GenBank accession	ID	Ho-roots-DAI2[1]	Ho-roots-DAI6	Ho-roots-DAI20	Mo-roots-DAI2[2]	Mo-roots-DAI6
elf1α	Eukaryotic elongation factor 1-alpha	AK061464	LOC_Os03g08050	169.862	182.981	218.46	167.042	176.264
eIF4a	Eukaryotic initiation factor 4a	AK073620	LOC_Os02g05330	65.5388	73.289	74.1441	83.4178	83.9693
SFua2	Splicing factor U2af	AK070198	LOC_Os05g48960	60.0201	60.818	87.201	67.6524	72.014
GAPDH	Glyceraldehyde-3-phosphate dehydrogenase	AK064960	LOC_Os04g40950	667.029	348.337	216.637	418.456	392.486
UBC1	Ubiquitin-conjugating enzyme E2	AK059694	LOC_Os02g42314	147.132	137.6	76.6485	164.072	102.718
PK	3-phosphoinositide-dependent protein kinase-1	AK073948	LOC_Os06g48970	19.8278	29.1471	33.0738	24.7674	21.7284
NBP	Nucleic acid binding protein	AK065717	LOC_Os06g11170	43.7882	60.9218	70.8847	51.8572	48.3598

[1]Ho-roots-DAI2, Ho-roots-DAI6, Ho-roots-DAI20 refer to transcripts expressed by *H. oryzae*-challenged rice roots at 2, 6 and 20 days after inoculation, respectively.

[2]Mo-roots-DAI2 and Mo-roots-DAI6 refer to transcripts expressed by *M. oryzae*-challenged rice roots at 2 and 6 days after inoculation.

note: Gene expressions were quantified by FPKM in RNA-seq analysis; FPKM:fragments per kilobase of exon model per million mapped reads.

Supplementary Table S6. KEGG enrichment analysis of DEGs of rice roots infected by *H.* or *M. oryzae* at different colonization stages.

Pathway ID	P-value[1]	Gene counts[2]	Description
**Ho-roots-DAI6-DAI2 enriched KEGG terms[3]**			
map00940	7.12E-09	14	lignin biosynthesis
map00620	0.007825776	6	Pyruvate metabolism
map00910	0.000498992	5	Nitrogen metabolism
map00270	0.000699872	8	Cysteine and methionine metabolism
map00480	5.48E-06	10	Glutathione metabolism
map00710	0.010024043	6	Carbon fixation in photosynthetic organisms
map04626	0.006556537	8	Plant-pathogen interaction
map05133	0.044987306	3	Pertussis
map04540	3.27E-06	6	Gap junction
map01120	2.69E-08	21	Microbial metabolism in diverse environments
map00061	0.000498992	5	sat-fasyn
map04145	0.000699872	8	Phagosome
map00051	0.000127574	7	Fructose and mannose metabolism
map00520	0.0037215	8	Amino sugar and nucleotide sugar metabolism
map00010	0.002002351	8	Glycolysis / Gluconeogenesis
map00680	5.63E-05	10	Methane metabolism
map01110	0	44	Biosynthesis of secondary metabolites
map00500	0.000315996	10	Starch and sucrose metabolism
map00980	8.32E-07	8	Metabolism of xenobiotics by cytochrome P450
map05010	0.010503277	7	Alzheimer's disease
map00360	9.58E-07	11	Phenylalanine metabolism
map01040	0.003196473	5	Biosynthesis of unsaturated fatty acids
map00630	0.00010297	6	Glyoxylate and dicarboxylate metabolism
map00982	8.32E-07	8	Drug Metabolism
map05130	1.21E-06	6	Pathogenic Escherichia coli infection
map01100	0	79	Metabolic pathways
**Ho-roots-DAI20-DAI6 enriched KEGG terms**			
map00940	0.041419229	8	lignin biosynthesis
map05323	0.017981279	4	Rheumatoid arthritis
map00196	4.77E-12	9	Photosynthesis - antenna proteins
map04966	0.01037324	4	Collecting duct acid secretion
map05012	0.000134337	10	Parkinson's disease
map05016	0.003650652	10	Huntington's disease
map00960	0.004011804	3	Tropane, piperidine and pyridine alkaloid biosynthesis
map00900	1.15E-05	8	Terpenoid backbone biosynthesis
map03010	1.20E-05	24	Ribosome
map00270	0.007780558	8	Cysteine and methionine metabolism
map05110	0.001618497	6	Vibrio cholerae infection

map00904	0.000264335	5	Diterpenoid biosynthesis
map00350	0.007660668	5	Tyrosine metabolism
map03040	0.00570304	13	Spliceosome
map04075	0.001479396	13	Plant hormone signal transduction
map04145	0.000156985	10	Phagosome
map01110	4.54E-11	45	Biosynthesis of secondary metabolites
map05010	0.000377481	10	Alzheimer's disease
map00190	1.47E-05	16	Oxidative phosphorylation
map00195	4.85E-06	14	Photosynthesis
map04142	0.000667966	7	Lysosome
map05120	0.003721812	5	Epithelial cell signaling in Helicobacter pylori infection
map01100	0	88	Metabolic pathways

[1]All P values were corrected for false discoveries due to multiple hypthosis testing using the Benjamin-Hochberg procedure.

[2]Total Gene numbers in significantly different expression.

[3]Ho-roots-DAI6-DAI2 refers to DEGs of *H. oryzae*-challenged roots (Ho-roots) at 6 days afer inoculation (DAI6) when compared to those of DAI2.

Supplementary Table S7. Genes and primer pairs used for qRT-PCR. Gene IDs are corresponded to Supplementary Fig. S4.

Gene_ID	Locus	Primer (Forward, 5'-3')	Primer (Reverse, 5'-3')
**House-keeping gene**			
1	LOC_Os03g08050	GAAGACGCACATCAACATCG	ACCTCTTGTTCATCTCAGCG
2	LOC_Os02g05330	TTTGGCAGGAAGGGAGTTG	GAACCTCTGGATGTCGAACAG
3	LOC_Os02g42314	GTGGACTGCTCTTATCAAGGG	GTCTTGAAATGCACATTCGGG
4	LOC_Os06g48970	TCTCTGGAAGCAAATCGACG	CAGATAAGGCAGTGGATACGG
**Genes involved in the starch degradation, glycolysis and TCA pathways**			
5	LOC_Os10g32810	GAGATGGAGAAGGACAACGAC	GAGGGAGATGTACTCGAAGTTG
6	LOC_Os10g41550	ATCCAGGTCTACTCCGACTAC	CATTGGCCTCAGGATACGAAG
7	LOC_Os10g17650	GTAACGCTCTATCACTGGGAC	TTCACCCTGTCCCCAAATG
8	LOC_Os03g50480	CAGCCTCATTACCTCCAGAAC	ATCTGAACAGCATCCTTCGAG
9	LOC_Os01g66940	GTCCCATCCTACAAAGTACAGC	TGGCAAATTTAATCGCTTCCTC
10	LOC_Os02g41590	CCTTCCTCACCAAGTATGACG	CCATTGAGCAACCCTGATAGAG
11	LOC_Os02g48360	CTAATGTCCCCGATGCTACTG	GTTTGCTGTTTGGGTTGTGAG
12	LOC_Os05g10650	GACAACGTCTACTGCTCGC	TGTTCTGCTTCTCCGTGATC
13	LOC_Os05g33380	AGGCGGAGAACATTGAGAAG	ACAGCGTCACCCTTGTATG
14	LOC_Os01g67860	ACCAACAAAGAGACCACCAC	GTCAATGGAAAGCTGCGATG
15	LOC_Os02g38920	ACCTTGTTTCCACTGACTTCC	TGCTGTATCCCCATTCGTTG
16	LOC_Os04g43090	TGGATGCGGATGAAACTGATG	CTTGCTCGAAAAGATTGGCTC
17	LOC_Os12g08170	GACGTTCAGAGTCGGTTACAC	ACACCCTTCGGCTTAATCTTC
18	LOC_Os05g49880	ACCTCTTCAACATCAACGCC	TTCTTGAACACCTCCGCTG
**Genes involved in lignin biosynthesis**			
19	LOC_Os08g34280	AAGATGTCGAACCAGAAGCTC	CACTTCACCGTCTCGTACAG
20	LOC_Os03g02939	ACTTCCATGACTGCTTCGTC	CTCAGGCTCTTGTTCGGTATC

21	LOC_Os03g13200	CGACAATAACCTCCCCAGC	TCACGAACTGGCACTGC
**Diversely expressed genes responding to biotic or abiotic stress in the *M. and H. oryzae*-challenged roots**			
22	LOC_Os11g47500	TCAAGGAAAGAGGCAGAACG	GAGAGGTCGAGCCAGTATTTG
23	LOC_Os04g41680	ATGAGACCGGACATATGTGC	GCCCGTAGTTGTAGTTCCAC
24	LOC_Os11g26760	CAGTTCCAGCCGGTGAAG	CTTGATTCCCTTCTTCCTCCTC
25	LOC_Os04g54810	GACTGGAAGCTGAACGGATAC	AGGGTTCTTGGTGTAGTGTTG
26	LOC_Os01g71080	TCTCCCCGGATACTTCATCTC	CTCTTGGGTGTAGAGCTGC
